# Constraining Polymers into β-Turns: Miscibility and Phase Segregation Effects in Lipid Monolayers

**DOI:** 10.3390/polym9080369

**Published:** 2017-08-17

**Authors:** Stefanie Deike, Marlen Malke, Bob-Dan Lechner, Wolfgang H. Binder

**Affiliations:** 1 Faculty of Natural Science II, Martin Luther University Halle-Wittenberg, Von-Danckelmann-Platz 4, 06120 Halle (Saale), Germany; stefanie.deike@chemie.uni-halle.de (S.D.); marlen.malke@iap.fraunhofer.de (M.M.); B.Lechner@exeter.ac.uk (B.-D.L.); 2 School of Physics, University of Exeter, Stocker Road, Exeter EX4, UK

**Keywords:** amphiphilic, copolymers, biomembranes, helical polymers, β-turns, lipid model membrane, Langmuir monolayer

## Abstract

Investigation of model biomembranes and their interactions with natural or synthetic macromolecules are of great interest to design membrane systems with specific properties such as drug-delivery. Here we study the behavior of amphiphilic β-turn mimetic polymer conjugates at the air–water interface and their interactions with lipid model membranes. For this endeavor we synthesized two different types of conjugates containing either hydrophobic polyisobutylene (PIB, *M*_n_ = 5000 g·mol^−1^) or helical poly(*n*-hexyl isocyanate) (PHIC, *M*_n_ = 4000 g·mol^−1^), both polymers being immiscible, whereas polyisobutylene as a hydrophobic polymer can incorporate into lipid membranes. The conjugates were investigated using Langmuir-film techniques coupled with epifluorescence microscopy and AFM (Atomic Force Microscopy), in addition to their phase behavior in mixed lipid/polymer membranes composed of DPPC (dipalmitoyl-*sn*-glycero-3-phosphocholine). It was found that the DPPC monolayers are strongly disturbed by the presence of the polymer conjugates and that domain formation of the polymer conjugates occurs at high surface pressures (π > 30 mN·m^−1^).

## 1. Introduction

Expanding the world of lipid bilayer membranes has led scientists towards polymer-based amphiphilic systems, in particular polymersomes [1,2], where the membrane is entirely made up of amphiphilic di- or triblockcopolymers. Being superior in mechanical performance, with significantly enhanced bending moduli in comparison to lipid-bilayer membranes [2], they also display drawbacks, such as a reduced membrane mobility, a large thickness, or significantly reduced transport properties across the membrane [3,4]. In order to merge the advantages of polymersomes with those of lipid bilayer membranes, mixed-systems between those two components have gained significant attention during the past years, placing the principles of formation, stability, and most of all miscibility of these two systems into the limelight [5,6,7,8].

The interaction of polymers with lipid membranes is strongly guided by immiscibility effects, exerted between the polymer segments by thermodynamic and steric constraints, in combination with the phase structure of the lipid membrane [6,7,8,9]. In general conduct, the mixture of polymers with lipids represents a polyphilic system [10,11,12,13], where several repulsive ordering principles are competing, such as the polymer with the same or another polymer, the polymer with the hydrophobic lipid tail or with the hydrophilic lipid head group. A strong interplay between ordering effects of the polymer (microphase segregation according to Flory-Huggins theory) is thus competing with liquid-crystallinity of the surrounding lipids, leading to “hybrid-vesicles”, often composed of individual domains of either component. Such mixed systems have been studied intensely, displaying biological effects via a modulation in membrane fluidity [14,15,16,17,18,19,20,21,22,23], or related to the enrichment of membrane proteins via phase segregation within hybrid lipid/polymer vesicles [24,25].

In the past, we have demonstrated studies [6,7,8,9,24,26,27,28,29,30,31,32,33,34,35], where mixed polymer/lipid mono- and bilayers had been investigated, both on Langmuir-monolayer systems [28] or within bilayer membranes [24,27,29,34], demonstrating that functionality and control of such domains can be achieved within the mono- and bilayer systems [27]. Thus functional biological recognition of gangliosides can be achieved specifically within/outside the lipid-domains [24], nanoparticles can be enriched selectively within the polymer domains [9,30,31,33], and supramolecular recognition leads to macroscopic effects on the vesicular surfaces via budding and fission of the lipid membrane [28].

**Figure 1:**
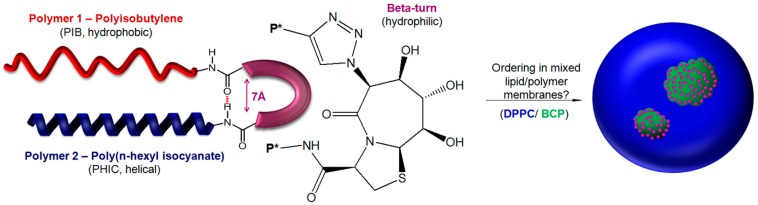
Concept of β-turn mimetic amphiphilic block copolymers and their incorporation into mixed membranes via folded polymer chains. (BCP = block copolymer).

Here, we describe the effects of an artificial polymer system on the domain-formation in lipid mono- and bilayers, based on an artificial β-turn, linking two different polymer chains (see Figure 1). Natural β-turns are folding elements in proteins, enabling the back-folding of a peptide chain via four amino acids. Besides peptidic β-turns, numerous synthetic low molecular weight structures have been found to mimic the folding in a defined manner [36,37,38,39]. The chosen β-turn mimetic is a purely geometrically constrained dipeptide from Geyer et al. (see Figure 1), where the distance between the azide functionality and the carboxylic moiety on the other side is only 7 Å, according to X-ray structure [40]. Moreover, its polyol structure is known for membrane affinity as well as interactions with membranes [41]. Due to the orthogonal groups (azide- and carboxyl-function), polymer chains can be attached on both sides of the turn, via azide/alkyne-“click” reaction and amidation reaction. Two different polymers—namely, hydrophobic polyisobutylene and helical poly(*n*-hexyl isocyanate)—have been chosen for the linkage. Depending on the attached polymer chain and the rigidity of the chains, differences in domain size and domain structure are expected after incorporation into a lipid mono- or bilayer membrane. Thus, in addition to the flexible polyisobutylene chains, we also investigated polyisocyanates that are known as rigid rod polymers, forming a stable 8_3_-helix with a rise of about 5.2 Å per turn which can incorporate into lipid bilayer membranes.

Dennison et al. [42,43] investigated the interaction between phospholipid monolayers and the *cis*- and *trans*-benzanilide, suggesting a strong dependence between the conformation and membrane binding driven by amphiphilicity. Similar to their findings that the *cis*-form penetrates stronger into the lipid system, we expect a folding of the polymer chains into the lipid membrane, supported by the conformational constraint of the β-turn.

## 2. Materials and Methods 

### 2.1. Materials

(Benzotriazol-1-yloxy) tripyrrolidinophosphonium hexafluorophosphate (PyBOP, purity > 99%) was purchased from Merck (Darmstadt, Germany). *N*-methlymorpholine (NMM, purity > 99%) was obtained from VWR (Alfa Aesar, Karlsruhe, Germany). The following chemicals were purchased from Sigma Aldrich (Taufkirchen, Germany) and used as received unless otherwise stated: 1,2-dipalmitoyl-*sn*-glycero-3-phosphocholine (L-DPPC, purity > 99%), 1,2-dihexadecanoyl-*sn*-glycero-3-phosphoethanolamine-*N*-(lissamine rhodamine B sulfonyl) (Rh–DHPE, Invitrogen, purity > 95%), rhodamine-B (purity > 95%), sodium azide (purity > 99%), 3-bromo-1-propanol (purity > 97%), *N,N*-dicyclohexylcarbodiimide (DCC, purity > 99%), 4-(dimethylamino)pyridine (DMAP, purity > 99%), copper(I) iodide (CuI, purity > 98%), *N,N*-diisopropylethylamine (DIPEA, purity > 99%), and Tris(benzyltriazolylmethyl) amine (TBTA, purity >97%). 1-Azido-3-propylrhodamine ester was synthesized according to literature [44]. Rhodamine-labeled poly(*n*-hexyl isocyanate) (Rh-PHIC) was synthesized similar to a previous reported method (see Supplementary Materials, Scheme S1) [45].

### 2.2. Methods

Surface Pressure-Area Isotherms: The surface pressure ***π*** of monolayers of the pure compounds and of different binary mixed systems of the PIBs and DPPC at the air-water interface via Langmuir film technique were performed using a Langmuir trough system (KSV, Helsinki, Finland) with a maximum available surface of 76,800 mm^2^. The investigated mixture of PIB or PHIC and DPPC was dissolved in chloroform (HPLC grade, Sigma Aldrich, St. Louis, MO, USA) at a concentration of 1 mM and the chloroform evaporated after applying the film. The monolayer was compressed at a barrier speed of 5 mm/min^−1^ and was started 15 min after spreading, to ensure the full evaporation of the solvent and a uniform monolayer formation.

Epifluorescence Microscopy: Imaging of monolayers at the air–water interface was performed using an “Axio Scope A1 Vario” epifluorescence microscope (Carl Zeiss MicroImaging, Jena, Germany) with a Langmuir Teflon trough (maximum area 264 cm^2^, two movable barriers; Riegler & Kirstein GmbH, Berlin, Germany). The trough was mounted on an x-y stage (Marzhauser, Wetzlar, Germany) with x-y-z motion control (Mac5000 system, Ludl Electronic Products, Hawthorne, NY, USA). The air-water surface was imaged by a 100 W mercury arc lamp, a long-distance objective (LD EC Epiplan-NEOFLUAR 50×) was used and the respective wavelengths were selected with a filter/beam splitter combination. An excitation wavelength of 557 nm and an emission wavelength of 571 nm were used with the appropriate Zeiss filter set (filter set 20, green light). The fluorescence images were taken during compression with a speed of 2 Å^2^·molecule^−1^·min^−1^ and recorded using an EM-CCD camera (ImageEM C9100-13, Hamamatsu, Herrsching, Germany). The analysis and data acquisition were done using AxioVision software (Carl Zeiss MicroImaging, Jena, Germany). Monolayer films of pure or mixed compounds in different molar ratios were prepared with a total spreading concentration of 1 mM in chloroform (HPLC-grade, Carl Roth, Karlsruhe, Germany) and fluorescently labeled Rh-DHPE or Rh-PHIC (0.01 mol %) were added to the stock solution. A defined volume of the solution was then spread on the water surface and, after 15 min, the compression was started.

Atomic Force Microscopy: The deposition of polymer films formed at air–water interface was carried out on silicon wafer. The surface of the silicon wafer was cleaned directly before using. They were placed in a Helmanex solution (1% in H_2_O) at about 50 °C for 10 min using ultrasound, rinsed with Helmanex solution, and then rinsed with double deionized water. This washing procedure was repeated, using CHCl_3_ and H_2_O. In the last step they were cleaned using a plasma-cleaner. The film deposition was performed through the vertical dipping method (Langmuir-Blodgett transfer) using a constant transfer rate of 2 cm·min^−1^. Finally, the LB films were dried in a desiccator at room temperature for 24 h. The surface morphology of LB films was investigated using a Multimode V AFM with Nanoscope VII Controller (Bruker, Santa Barbara, CA, USA) coupled with reflected light microscopy (Olympus MM10,Olympus Europa Holding GmbH, Hamburg, Germany). The images were obtained in tapping mode using TESPA cantilever (NanoAndMore GmbH, Wetzlar, Germany).

### 2.3. General Procedure for the Synthesis of β-Turn Mimetic Conjugates

**Scheme 1:**
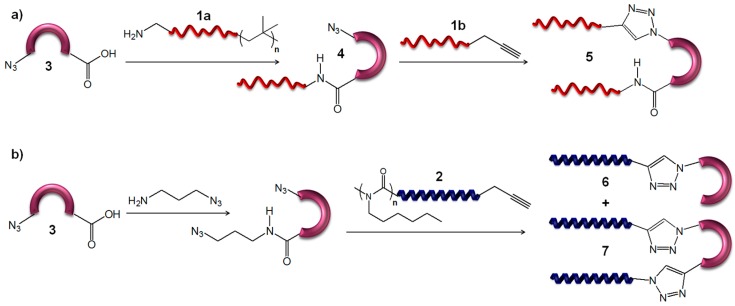
Synthesis of (**a**) β-turn mimetic polyisobutylene conjugates **4** and **5**, (**b**) β-turn mimetic poly(*n*-hexyl isocyanate) conjugates **6** and **7**.

Heterotelechelic (α-ω-functionalized) PIBs bearing either an amine moiety (compound **1a**, *M*_n_ = 5000 g·mol^−1^, *PDI* = 1.30) or an alkyne moiety (compound **1b**, *M*_n_ = 4700 g·mol^−1^; *PDI* = 1.39) were obtained via living carbocationic polymerization (LCCP) of isobutylene by combining the synthetic methods of Olubummo et al. [46] and Morgan et al. [47]. One-arm PIB conjugate **4** was synthesized by amidation reaction of amine functional PIB **1a** and the carboxylic acid of BTD **3** using (benzotriazol-1-yloxy)-tripyrrolidinophosphonium hexafluorophosphate (PyBOP, 1.4 equiv) and *N*-methyl morpholine (NMM, 2.0 equiv) as coupling reagents (Scheme 1a). The two-arm PIB conjugate **5** was obtained by azide/alkyne “click” reaction of **1b** and **4** using CuSO_4_·5H_2_O (0.2 equiv), sodium ascorbate (0.2 equiv), DIPEA (1.0 equiv), and TBTA (0.1 equiv) (Scheme 1a) [35].

The syntheses of β-turn mimetic poly(*n*-hexyl isocyanate) (PHIC) conjugates **6** (*M*_n_ = 4500 g·mol^−^^1^; *PDI* = 1.07) and **7** (*M*_n_ = 7900 g·mol^−^^1^; *PDI* = 1.14) have been previously published by our group (Scheme 1b) [45]. Detailed chemical structures of the conjugates are given in Figure S3 (see Supplementary Materials). 

## 3. Results and Discussion

### 3.1. Polyisobutylene Conjugates

#### 3.1.1. π-A Isotherms

**Figure 2:**
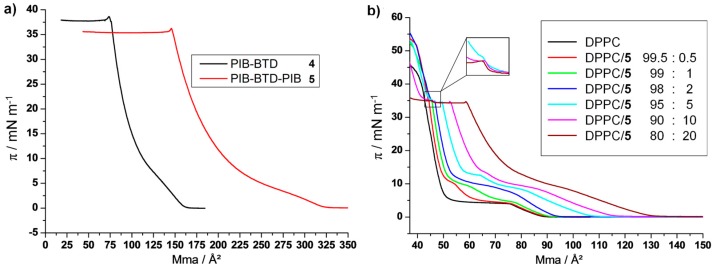
π-A isotherms of (**a**) the pure PIBs **4** and **5** and (**b**) DPPC/**5** mixtures at 20 °C. The black curve represents the isotherm of the pure DPPC and the different colored curves represent the different molar ratios of the DPPC/polymer mixtures.

Monolayer compression isotherms were conducted for the pure β-turn mimetic PIB conjugates **4** and **5** (Scheme 1), and for the DPPC/polymer mixtures with different molar ratios (Figure 2). The π-A isotherms of pure PIB-BTD **4** and PIB-BTD-PIB **5** monolayers show the lift-off at 161 Å^2^ and 322 Å^2^ respectively and reach a collapse pressure of 37 mN·m^−1^ (73 Å^2^) and 35 mN·m^−1^ (145.0 Å^2^). The doubling of the mean molecular area (mma) value is in exact agreement with the double molecular weight of **5** in comparison to **4**, implying that the β-turn as such plays only a minor influence on the molecular ordering of the PIB chains. The small tips at the beginning of the plateaus mark a metastable state and the following single plateau with a quite constant surface pressure indicates a so-called roll-over collapse leading to multilayer formation. This phenomenon has been previously observed for similar amphiphilic block copolymers with short hydrophilic blocks [48,49,50].

To investigate the folding of β-turn mimetic PIB conjugate **5** when incorporated into a membrane, Langmuir monolayer measurements were conducted, which serves as a model for roughly half a bilayer membrane. Therefore, mixed hybrid membranes composed of DPPC and PIB with different ratios of DPPC/PIB were investigated. As comparison for folding effects, the single strand conjugate **4**, where only one PIB strand is connected to the β-turn mimetic BTD, was also investigated. Due to the amphiphilic structure of **4** and **5**, the hydrophilic β-turn mimetic BTD should serve as anchor in the water phase, whereas the hydrophobic PIB chains should orient along the air–water interface. Figure 2b shows the monolayer isotherms of the DPPC/**5** mixtures with 0.5–20 mol % content of **5**. The lift-off areas of the mixtures are shifted to higher mean molecular areas with a higher content of **5**. However, the experimental values of the lift-off area are smaller than the expected ones, and thus attractive lipid/polymer interactions are concluded. This behavior was already observed in our group for PIB-PEO copolymers [29]. In contrast to previous work and to the isotherms of the pure compounds, two plateaus could be observed during compression, whereby with increasing content of **5** the surface pressures for these plateaus increase as well. 

As the transition state of the lipid monolayer (LE/LC, first plateau) is at a higher pressure (up to 10 mN·m^−1^) in comparison to pure DPPC, the PIB-BTD-PIB conjugate **5** strengthens the expanded phase, presumably because the amphiphilic polymer molecules disturb the rearrangement of the lipid molecules at the air–water interface due to their partial miscibility with the LE phase of the DPPC, thus hindering the lipid packing. The second plateau (up to 15 mN·m^−1^) gives a hint for a second transition, what we believe is the new arrangement/ordering of the polymer molecules, meaning that the apolar PIB chains straighten up, but are still tilted. This could be confirmed by further compression due to the existence of the previously titled “collapse” plateau (π = 35 mN·m^−1^) where the polymer molecules lift up from the subphase to build upper layers or squeeze out, whereby the length of this plateau is directly correlated to the mol fraction of **5**. After this plateau, the behavior of the isotherms only depends on the DPPC molecules, and the pressure of the isotherms increases drastically up to 58 mN·m^−1^ at which point the whole monolayer collapses. This region is known as the transition of the lipid molecules from the liquid condensed state to the solid phase state [51]. Up to this point, similar isotherm behavior could be observed for the DPPC/**4** mixtures with 1.0–30 mol % content of **4** (Figure S5, Supplementary Materials) and it seems that there are no perceptible differences whether one PIB chain is linked to the β-turn mimetic BTD or two PIB chains are linked to the same BTD molecule. 

Having a deeper look into the “collapse” plateau (π = 35 mN·m^−1^), a step at an area of 45.5 Å^2^ per molecule within the plateau is visible (Figure 2b). This area is in exact agreement with the calculated area (45 Å^2^) occupied by one molecule of BTD in a liquid condensed monolayer. Therefore, the third transition can be clearly attributed to the presence of the two PIB chains on the same β-turn mimetic BTD and marks a transition within the β-turn mimetic PIB conjugate **5** which is the ordering of the chains along one β-turn mimetic BTD molecule.

#### 3.1.2. Epifluorescence Microscopy

**Figure 3:**
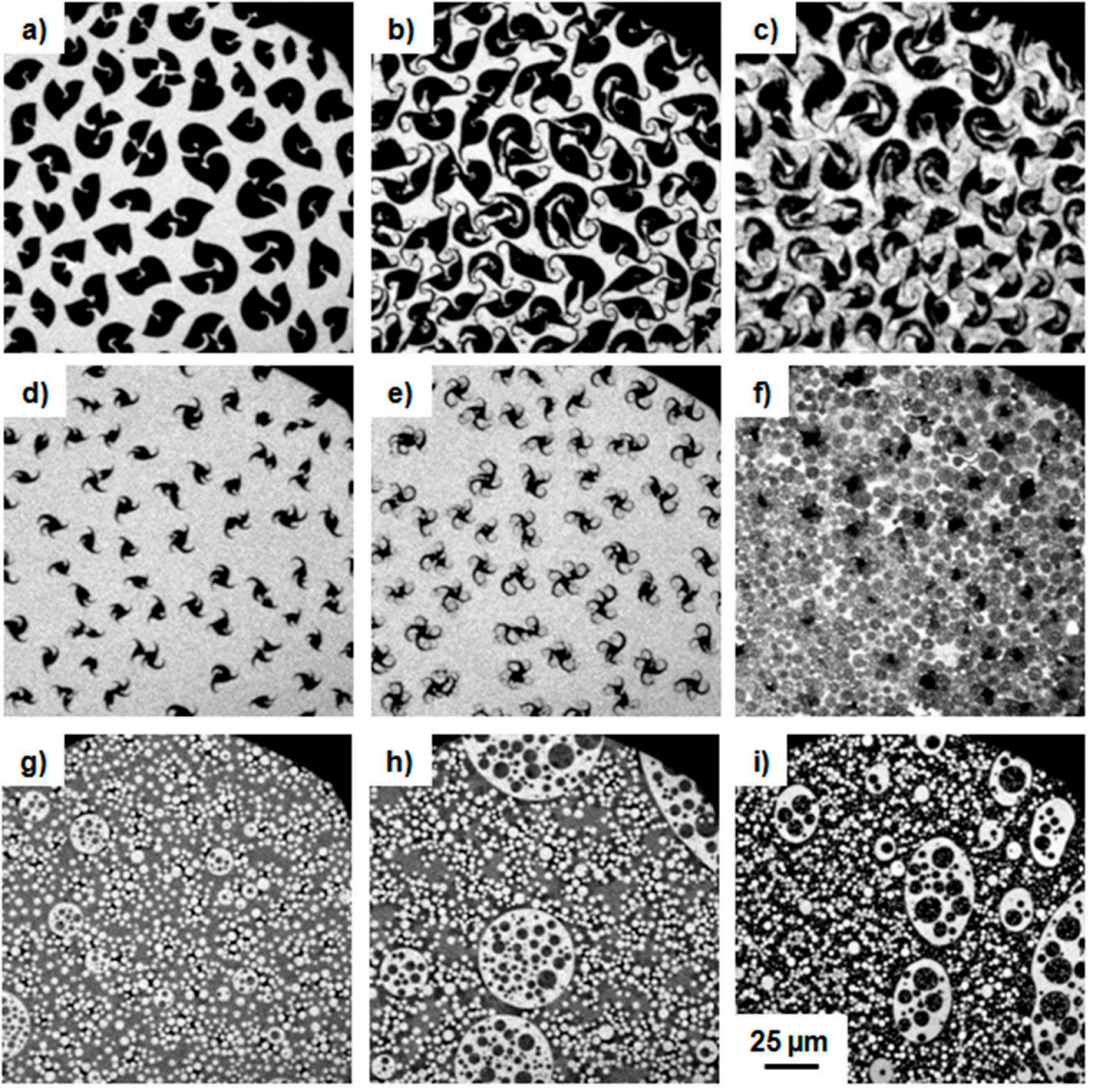
Epifluorescence microscopy images of monolayers of DPPC/**5** with different molar ratios (**a**–**c**) 99.5:0.5, (**d**–**f**) 99:1, and (**g**–**i**) 90:10 at the air–water interface at 20 °C. The images were recorded at constant compression of the spread monolayer in the region of the second transition state (second plateau) at the following surface pressures: (**a**) 5.8, (**b**) 7.2, (**c**) 7.6, (**d**) 6.6, (**e**) 6.9, (**f**) 9.7, (**g**) 9.4, (**h**)10.3, and (**i**) 29.2 mN·m^−^^1^.

Figure 3 shows the epifluorescence images of the monolayers of DPPC/**5** with different molar ratios (a–c) 99.5:0.5, (d–f) 99:1, and (g–i) 90:10 in the region of the second transition. The image of the first ratio (99.5:0.5) taken at a pressure of 5.8 mN·m^−1^ (Figure 3a) is relatively similar to pure L-DPPC (see Supplementary Materials, Figure S6), which possesses propeller like domains with a chirality-dependent curling direction to the left. An equivalent image was obtained for the one-arm conjugate **4** using 1 mol % (see Supplementary Materials, Figure S7). In contrast to the propeller domains of pure L-DPPC, the propeller structures are irregular and not so compact, which indicates a non-equilibrium behavior in the monolayer [51] and substantiates the coexistence of different phases within the first plateau. 

With increasing content of the amphiphilic PIB **5** and progressing compression, a thinning of the propeller tips and spiral formation can be observed. This phenomenon was already observed for DPPC co-spread with cholesterol or perfluorinated PGMA and could be explained by the reduced line tension, due to the formation of a new LC DPPC/polymer/LE DPPC boundary. Therefore, a partial miscibility of the amphiphilic PIB **5** with the LE phase of DPPC could be concluded [52]. Due to further compression, the miscibility increases, the phase boundaries begin to vanish, and the dye is forced into the LC phase (Figure 3f). Figure 3g,h show the images of the compression isotherm with 10 mol % of **5**. In contrast to the previous measurements using just a small content of **5** no propeller domains are visible (Figure 3g, 9.4 mN·m^−1^). The first black domains (LC DPPC) appear at the boundaries to the bright round LE domains, which differ in their size and can include smaller round grey domains. Moreover, the black LC domains seem to bridge the bright domains. With increasing content of **5** (see Supplementary Materials, Figure S8) the bright domains increase as well. Therefore, they could be assigned to the polymer rich LE phase, and the grey background constitutes the lipid rich LE phase. Due to further compression, the dark LC domains grow and stronger clustering between the bright LE domains and the dark LC domains could be observed (Figure 3g, 10.3 mN·m^−1^). While continuing compression, the previously round shaped domains become oval and the contrast between dark and bright increases together with a shrinkage of the single domain size. Even here, the dye is forced into the ordered LC domain as bright small dots appear out of the dark background Figure 3h, 29.2 mN·m^−1^). 

The one-arm conjugate **4** showed comparable results (see Supplementary Materials, Figure S7). Imaging of the third plateau was not possible using epifluorescence microscopy, as no differences according to contrast, shape, and size of the single domains could be observed at pressures higher than 20 mN·m^−1^. 

#### 3.1.3. AFM

**Figure 4:**
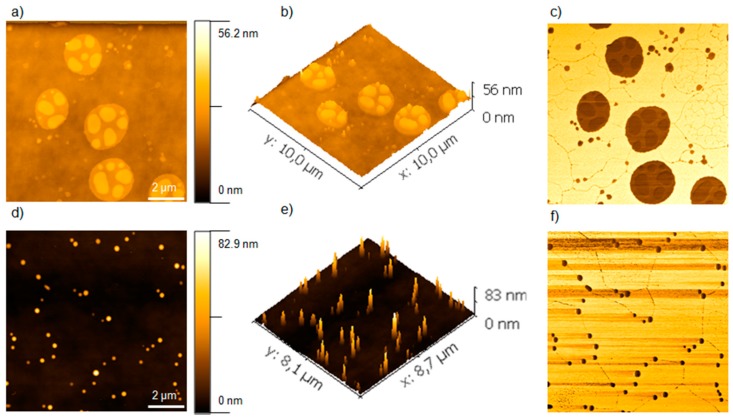
AFM topography (**a**, **b**, **d,** and **e**) and phase contrast images (**c** and **f**) of mixed monolayers of DPPC/**5** 90:10 mol % transferred at surface pressures of (**a**–**c**) 30 mN·m^−^^1^ and (**d**–**f**) 40 mN·m^−^^1^, respectively.

To understand the molecular organization of mixed DPPC/**5** monolayers at higher surface pressures, we conducted AFM measurements using the Langmuir Blodgett (LB) technique for a mixture of 90:10. As the region within the third plateau is the coexistence of two phases/states, one sample before the plateau (30 mN·m^−1^) and one sample after the plateau (40 mN·m^−1^) were measured, where the pressure 30 mN·m^−1^ is comparable to the lateral pressure of biological membranes [53]. Figure 4 shows the AFM topography (a, b, d, and e) and phase contrast images (c and f) of monolayer of DPPC/**5** 90:10 mol % transferred at surface pressures of (a–c) 30 mN·m^−1^ and (d–f) 40 mN·m^−1^. As the topography images show no defects, a complete transfer from the monolayer onto the Si-wafer can be concluded. The height images of DPPC/**5** (90:10 mol %), transferred at a surface pressures of 30 mN·m^−1^ (Figure 4a,b), show randomly distributed cylindrical PIB domains with a dominating height of ~15 nm out of the plane lipid monolayer (LC domain) or out of round shaped plateaus (height ~5 nm, due to partial squeeze out of the PIB), thus displaying three different domains. These cylindrical structures are in fact disc-like domains considering their dimensions, whereby their height correlates with a single-folded PIB chain [28,48]. Also, the phase image (Figure 4c) shows the three different phases, proving that the PIB discs are squeezed out (dark: soft) of the PIB rich plateau, which are surrounded by the LC phase of the lipid (bright: hard, due to the small thickness and the underlying silicon). The plane lipid domain is cross cut by a network of small paths, together with small dark dots lined up as a string of pearls, which are only visible in the phase image. As these network and dots appear dark, they are softer than the surrounding LC phase of the lipid and show the previously mentioned newly formed LC DPPC/polymer/LE DPPC boundary, which substantiates the hypothesis of the partial mixing of the polymer with the lipid at this interface.

With increasing transfer pressure to 40 mN·m^−1^ (Figure 4d–f), the PIB plateaus vanished and cone like PIB domains out of the plane lipid LC domains are formed. These structures, appearing here as cylinders (see Figure 4e), have a dominated height of 40 nm (maximum height of 80 nm), indicating the further squeezed out polymer **5**. With increasing pressure, these domains are decrease in surface area, but increase in height. As visible in the phase image (Figure 4f), they appear as dark lines at the phase boundary. Thus, the amphiphilic PIB **5** is forced into the lipid domains and subsequently squeezed out. Investigations of the one-arm conjugate **4** results in similar results (see Supplementary Materials, Figure S9). Only, the maximum height reached for the cone-like PIB domains is smaller (60 nm) compared to the two-arm conjugate **5** (80 nm). Thus, the linkage of two chains onto the β-turn mimetic structure provides an increased stabilizing effect.

### 3.2. Poly(n-Hexyl Isocyanate) Conjugates

The surface properties of pure PHIC [54,55,56,57] and of copolymers with poly(2-vinyl pyridine), polyisoprene or polystyrene [58,59] have been previously investigated. Kawaguchi et al. [54] found that pure PHIC forms a stable film on the air–water interface and comparison of experimental data with theoretical calculations suggested that the PHIC rods lay on the surface. Fluorescence microscopic images showed that the monolayer is in a liquid-like state and that formation of aggregates or pancakes occurs for PHIC of high molecular weight (>12 kDa). Once formed, these aggregates are difficult to deform upon expansion. Gargallo et al. [58] observed a second pseudoplateau for pure PHIC at a surface pressure of around 12 mN·m^−1^ and an area around 15 Å^2^ per repeating unit. They supposed that either a phase transition or the collapse of the monolayer is taking place. Hysteresis experiments for PHIC-PI and PHIC-PS copolymers suggested a collapse pressure of around 11 mN·m^−1^ at an area of 10 Å per repeating unit. Theoretical calculations supported these findings and suggested that two polymer chains start to extend toward the air at about 15 Å^2^ per repeating unit, which could result in the collapse of the monolayer due to destabilization. Beside these studies, copolymers with a more hydrophilic block than PHIC have not been investigated. Therefore, we were interested in the synthesis and investigation of β-turn mimetic PHIC conjugates, using a hydrophilic β-turn as an anchor to the air-water interface.

#### 3.2.1. π-A Isotherms

**Figure 5:**
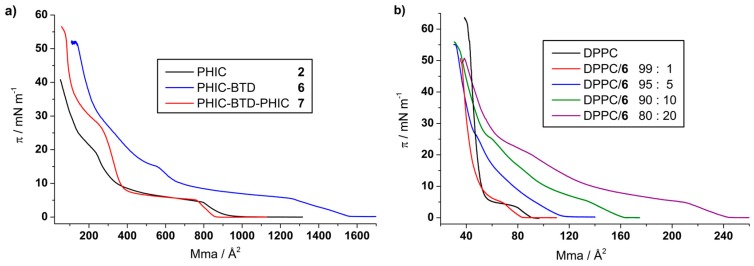
π-A isotherms of (**a**) the pure PHIC **2** and of β-turn mimetic conjugates **6** and **7** and of (**b**) DPPC/**6** mixtures at 20 °C. The black curve represents the isotherm of the pure DPPC and the different colored curves represent the different molar ratios of the DPPC/polymer mixtures.

Monolayer compression isotherms were conducted for pure PHIC **2** and for the conjugates **6** and **7** as well as for mixtures of **6** with DPPC in different molar ratios. Figure 5a shows the compression isotherms for PHIC **2** (black curve), PHIC-BTD **6** (red curve), and PHIC-BTD-PHIC **7** (blue curve). The isotherm of PHIC **2** shows as similar behavior as described in literature, with a lift-off at 905 Å^2^, corresponding to an area per repeating unit of 28.8 Å^2^. The one-arm conjugate **6** showed a lowering of the area of lift-off to 860 Å^2^ (25.0 Å^2^ per repeating unit), while the two-arm conjugate **7** showed a lift-off at 1560 Å^2^ (23.6 Å^2^ per repeating unit). This is approximately a doubling of the mean molecular area in regard to **6** and is thus in good agreement with the doubling of the molecular weight, as it has been seen before for the PIB conjugates. A shift of the transition to lower mean molecular area has previously been observed for PHICs with higher molecular weights due to the loss of rigidity [55] and for copolymers with PS and PI [58]. Thus, we assume that the β-turn mimetic structure leads to a higher packing density of the PHIC chains, which should lift out of the plane due to the hydrophilic anchor. For the two-arm conjugate, this is even more pronounced than for the one-arm conjugate, indicating an influence of the connection of two polymer chains to the same turn and thus a folding into restricted space.

The one-arm conjugate **6** shows an increase of the pressure for the second plateau in comparison to the homopolymer, with a steep increase in pressure afterwards, which is related to the high surface coverage indicating the condensed state. In contrast, the two-arm conjugate **7** shows a lowering of the second plateau and the appearance of a constant slope region between the plateau and the condensed state. This behavior has been previously observed for PS-PEO block copolymers and has been assigned to a quasi-brush regime in which some organization is taking place [60]. Thus, the β-turn mimetic structure should also lead to a higher organization due to the anchoring at the air–water interface. 

Figure 5b shows the monolayer isotherms of the DPPC/**6** mixtures with 1–20 mol % content of **6**. The mean molecular area of lift-off increases with increasing content of **6**. However, the experimental values of the lift-off area are smaller than the theoretical ones for low amounts of **6** (1–5 mol %), while they are equivalent at 10–20 mol %. Thus, for a low content of **6**, attractive lipid/polymer interactions can be concluded, similar to the PIB conjugates and PIB-PEO copolymers [29], while a higher content of **6** leads to the loss of attractive interactions, which might be related to domain formation of the PHIC chains. The addition of **6** to DPPC leads to a vanishing of the pronounced plateau of DPPC and the formation of a pseudo-plateau, indicating a hindering of the lipid packing, similar to the PIB conjugates, due to partial miscibility of the amphiphilic polymer with the LE phase of the lipid. At a content greater than 5 mol % of **6**, a second plateau is visible at higher surface pressures, which is at surface pressures close to the one of pure **6**. Consequently, this plateau can be assigned to some ordering of the polymer molecules. Further compression leads to a collapse of the monolayer, which takes place at lower surface pressures for the DPPC/**6** mixtures than for pure DPPC and occurs at a surface pressure close to the one of pure conjugate **6**. Thus, the polymer weakens the condensed phase of the lipid and enhances the monolayer collapse.

#### 3.2.2. Epifluorescence Microscopy

**Figure 6:**
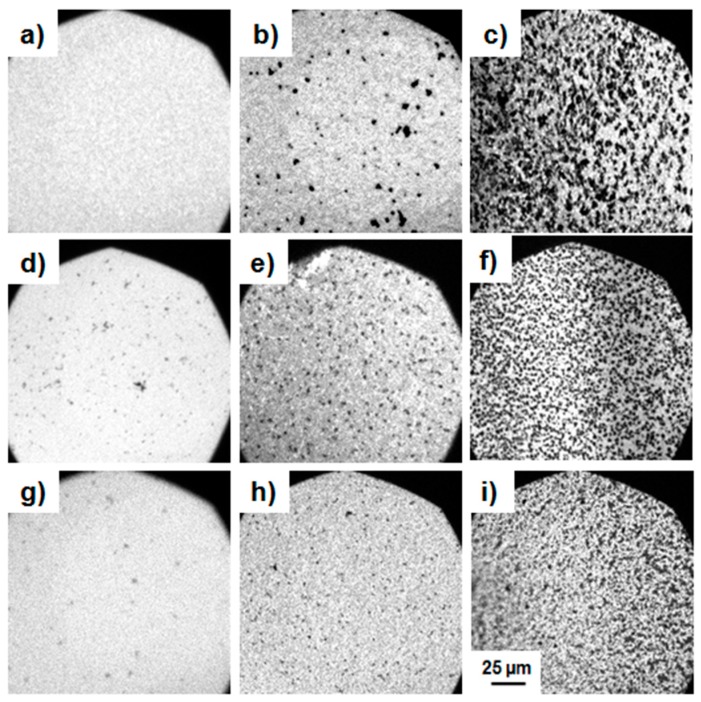
Epifluorescence microscopy images of monolayers of **2** (**a**–**c**), **6** (**d**–**f**), and **7** (**g**–**i**) at the air–water interface at 20 °C. The images were recorded at constant compression of the spread monolayer at the following surface pressures: (**a**) 4.3, (**b**) 21.0, (**c**) 28.4, (**d**) 5.0, (**e**) 15.6, (**f**) 34.4, (**g**) 0.1, (**h**) 9.2, and (**i**) 20.0 mN·m^−1^.

For previous fluorescence microscopy studies on PHIC films, a phospholipid dye with a completely different chemical structure than PHIC has been used and only measurements below the pressure of the first plateau have been conducted [54,55]. We synthesized a rhodamine-labeled PHIC to gain further insight into the behavior at surface pressures above the first plateau. Figure 6 shows the epifluorescence images of the monolayers of **2** (a–c), **6** (d–f), and **7** (g–i) around the first and second plateau.

At surface pressures below 5 mN·m^−1^ and before reaching the first plateau, a mostly bright surface was observed for all three polymers, indicating a homogenous distribution of the dye on the surface. Even at higher areas, no domain formation was observed, as it has been previously found using a phospholipid dye, thus indicating that the choice of dye plays a critical role in the epifluorescence measurements. At higher surface pressures, in the region after the first and before the second plateau, the dye is excluded from some parts and black domains become visible. These domains are less regular in shape and size for pure PHIC **2** (Figure 6b) than they are for the conjugates (Figure 6e,h). The amount of domains increases upon further compression above the second plateau (Figure 6c,f,i). While the size and distribution remains regular in case of the conjugates **6** and **7**, the pure PHIC **2** forms non-defined domains of different sizes. Thus, the attachment of a hydrophilic anchor enhances the ability of PHIC to form well-defined films at the air-water interface. 

**Figure 7:**
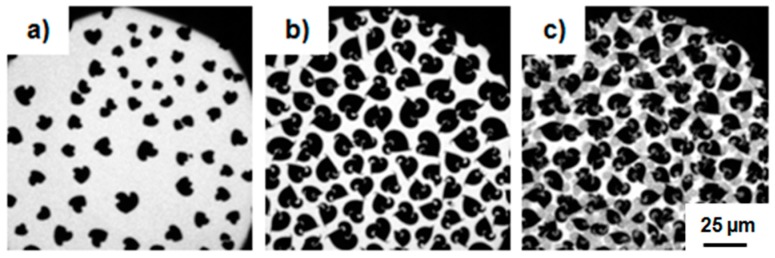
Epifluorescence microscopy images of monolayers of DPPC/**6** at a ratio of 99:1 at the air–water interface at 20 °C. The images were recorded at constant compression of the spread monolayer at the following surface pressures: (**a**) 5.3, (**b**) 7.3, (**c**) 7.9 mN·m^−^^1^.

Epifluorescence images of the monolayer were also taken using a mixture of DPPC and the conjugates **6**. Figure 7 shows one example of DPPC/**6** in a ratio of 99:1. As demonstrated before for the PIB conjugates, the propeller like structures of DPPC are visible and line thinning occurs upon compression. Compared to the PIB conjugates (Figure 3) the thinning is less pronounced, indicating a lower miscibility of the PHIC conjugates which confirms the results obtained from the compression isotherms. The images for β-turn mimetic conjugate **7** were similar (see Supplementary Materials, Figure S10). 

## 4. Conclusions

The behavior of β-turn mimetic amphiphilic copolymers at the air–water interface and their interactions with DPPC as lipid model membrane were investigated. Therefore, a hydrophilic dipeptide, serving as β-turn mimetic structure was linked to either one or two polymer chains. Two different types of polymers were used: either hydrophobic polyisobutylene (PIB) **1**, prepared by living carbocationic polymerization or helical poly(*n*-hexyl isocyanate) (PHIC) **2**, synthesized via titanium-catalyzed coordination/insertion polymerization. The conjugates containing PIB as hydrophobic chains showed a partial miscibility with DPPC, resulting in a shift of the lift-off in the π-A isotherms to lower areas for different mixtures and a reduced line tension visible from the epifluorescence images. For higher contents of the PIB conjugates, domains with different heights were formed as visible from AFM, indicating a squeeze out of PIB at a second plateau at 37 mN·m^−1^. The conjugate **5** bearing two polymer chains attached to the same β-turn showed a higher miscibility with DPPC and an improved stabilization of the formed domains in comparison to conjugate **4**, bearing only one polymer chain. 

Langmuir isotherms of helical PHIC conjugates **6** and **7** showed a lower lift-off than pure PHIC **2**, indicating attractive interactions and a denser organization due to the hydrophilic moiety. Epifluorescence images of the monolayer using a rhodamine-labeled PHIC as dye revealed the formation of domains of homogenous size and distribution at higher surface pressures for β-turn mimetic conjugates **6** and **7**, while inhomogeneities were observed for pure PHIC **2**. 

In summary, the attachment of a hydrophilic β-turn mimetic structure has a significant influence on the behavior of PIB and PHIC at the air–water interface, improving the ability to form stable films and leading to the formation of domains when incorporated into lipid model membranes of DPPC. 

## Supplementary Materials

The following are available online at www.mdpi.com/2073-4360/9/8/369/s1, Figure S1: ^1^H NMR spectrum of Rh-PHIC, Figure S2: MALDI-TOF MS of Rh-PHIC, Figure S3: Chemical structures of PIB **1a** and **1b** and of PIB conjugates **4** and **5**, Figure S4: Chemical structures of PHIC **2** and of PHIC conjugates **6** and **7**, Figure S5: π-A isotherms of DPPC/**4** mixtures, Figure S6: Epifluorescence microscopy images of L-DPPC monolayer, Figure S7: Epifluorescence microscopy images of monolayers of DPPC/**4** mixtures, Figure S8: Epifluorescence microscopy images of monolayers of DPPC/**5** 80:20, Figure S9:AFM of mixed monolayer of DPPC/**4**, Figure S10: Epifluorescence microscopy images of monolayers of DPPC/**7** 99.5:0.5.
