# Supplementary material for: Constraining Polymers into β-Turns: Miscibility and Phase Segregation Effects in Lipid Monolayers
Source: Polymers (Basel). 2017 Aug 17;9(8):369. doi: 10.3390/polym9080369 (PMC6418963; doi:10.3390/polym9080369)
Supplement: Supplementary file 1 [file polymers-09-00369-s001.pdf]

Supplementary Materials

Constraining polymers into beta-turns: miscibility and phase segregation effects in lipid monolayers

Stefanie Deike, Marlen Malke, Bob-Dan Lechner, Wolfgang H. Binder

1. Synthesis of 1-Azido-3-propylrhodamine ester (Rh-N_3)[1]

Rhodamine B (2.0 g, 4.2 mmol, 1.0 equiv) was dissolved in dry DCM (50 mL) under an argon atmosphere. 1-Azido-3-propanol (460 mg, 4.6 mmol, 1.1 equiv) and DMAP (48 mg, 0.4 mmol, 0.1 equiv) were added, and the solution was cooled to 0 °C. DCC (1.74 g, 8.4 mmol, 2.0 equiv) was added and the solution was stirred and warmed to room temperature overnight. The white precipitate was filtered off, DCM (50 mL) was added and the organic phase was washed with $NaHCO_3$ (2 x 100 mL) and water (100 mL). The organic phase was dried over sodium sulfate, filtered and the solvent was removed under vacuo. The resulting dark violet solid was purified using column chromatography (DCM/MeOH = 20:1; R_f = 0.2). After removal of the solvent, a dark violet solid was obtained.

^{1}H NMR ($CDCl_3$, 400 MHz) δ 8.28 (dd, *J* = 1.0, 7.9 Hz, 1H), 7.82 (dt, *J* = 7.7, 1.3 Hz, 1H), 7.74 (dt, *J* = 7.6, 1.3 Hz, 1H), 7.32 (dd, *J* = 7.5, 0.9 Hz, 1H), 7.07 (d, *J* = 9.8 Hz, 2H), 6.89 (d, *J* = 2.5 Hz, 1H), 6.87 (s, 3H), 4.12 (m, 2H), 3.63 (q, *J* = 7.2 Hz, 8H), 3.18 (t, *J* = 6.6 Hz, 2H), 1.71 (m, 2H), 1.32 (t, *J* = 7.1 Hz, 12H).
^{13}C NMR ($CDCl_3$, 100 MHz) δ 165.1, 158.7, 157.9, 155.7, 133.6, 133.3, 131.5, 131.3, 130.6, 130.5, 129.9, 114.4, 113.6, 96.7, 62.7, 48.1, 46.2, 28.1, 12.8.
ESI-TOF-MS: m/z = 526.2807 (Calc. 526.2818; $[M-Cl]^+$), 498.2505 (Calc.: 498.2505; $[M-Cl-C_2H_2]^+$), 471.2591 (Calc. 470.2192; $[M-Cl-(C_2H_2)_2]^+$).

2. Synthesis of rhodamine-labeled PHIC (Rh-PHIC)[2]

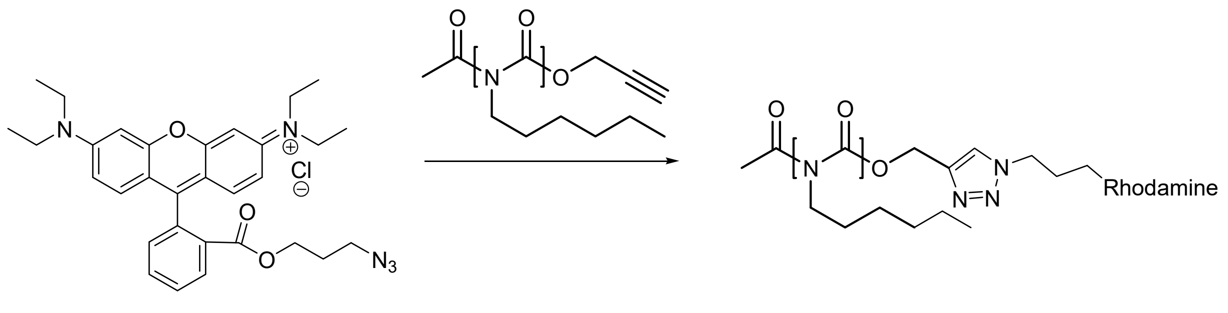

PHIC **2** (4.0 kDa, 44 µmol, 200.0 mg) and 1-azido-3-propylrhodamine ester (1.2 equiv, 53 µmol, 29.7 mg) were weighed into a Schlenk-flask and dissolved in THF (4.0 mL). The solution was degassed by bubbling with nitrogen for 30 minutes. CuI (0.1 equiv, 4.4 µmol, 0.9 mg) and DIPEA (2.0 equiv, 15.5 µmol, 0.088 mmol) were added under a counterflow of nitrogen and the solution was further degassed for 15 minutes. The reaction mixture was stirred at 35 °C for 24 hours. Afterwards, the solvent was removed under vacuo and the crude product was purified by silica gel column chromatography starting with $CHCl_3$/MeOH = 100:1 to remove non-functionalized PHIC and then changing to 20:1 to obtain the final product as a dark violet solid.

^1H NMR ($CDCl_3$, 400 MHz) δ 8.30 (s, 1H, H_7), 7.77 (m, 2H, H_8, H_9), 7.29 (m, 1H, H_{10}), 7.12 (d, *J* = 9.5 Hz, 2H, H_{11}), 6.90 (dd, *J* = 9.5, 2.3 Hz, 2H, H_{12}), 6.83 (d, *J* = 2.4 Hz, 2H, H_{13}), 5.29 (s, 2H, H_2), 4.38 (s, 2H, H_6), 4.13 (t, *J* = 6.0 Hz, 2H, H_4), 3.69 (s, H_d), 3.62 (m, 8H, H_{14}), 2.27 (s, 3H, H_1), 2.14 (dt, *J* = 13.1, 9.5 Hz, 2H, H_5), 1.61 (s, br, H_c), 1.31 (s, br, H_b), 1.16 (t, *J* = 7.1 Hz, 12H, H_{15}), 0.87 (s, br, H_a).

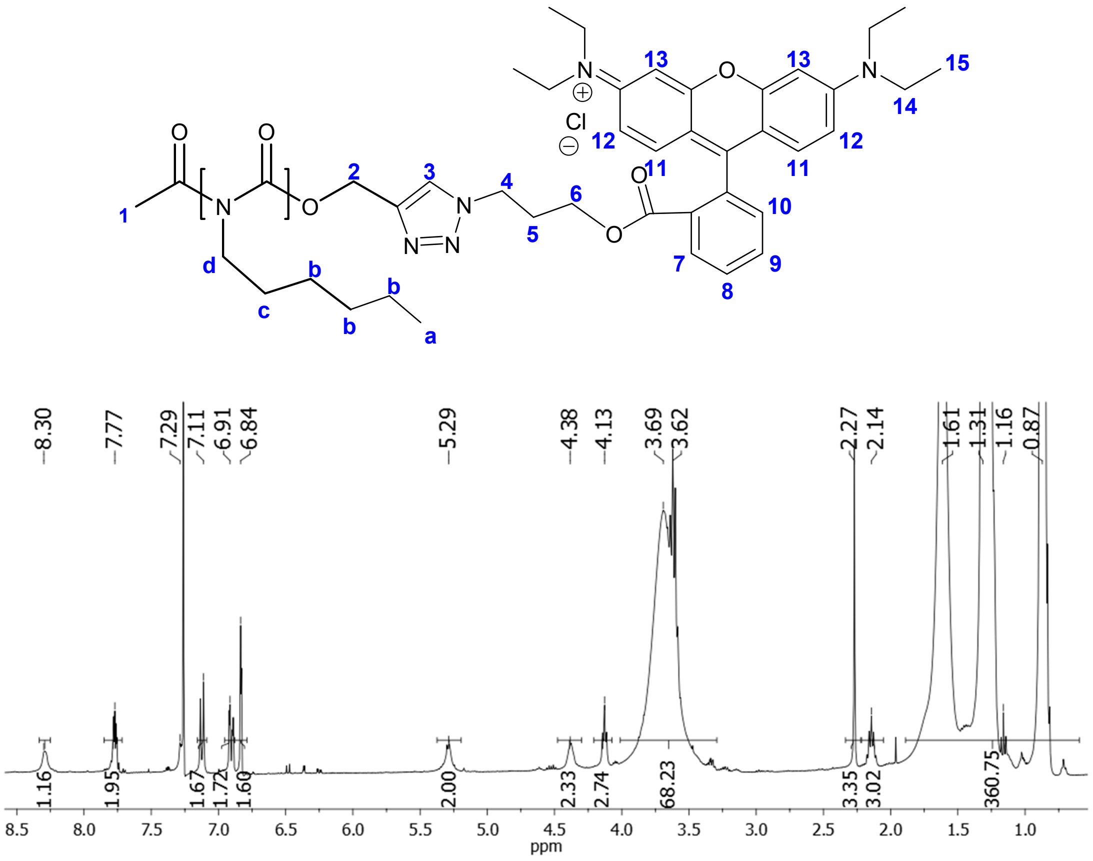

Figure S1. ^1H NMR spectrum of Rh-PHIC.

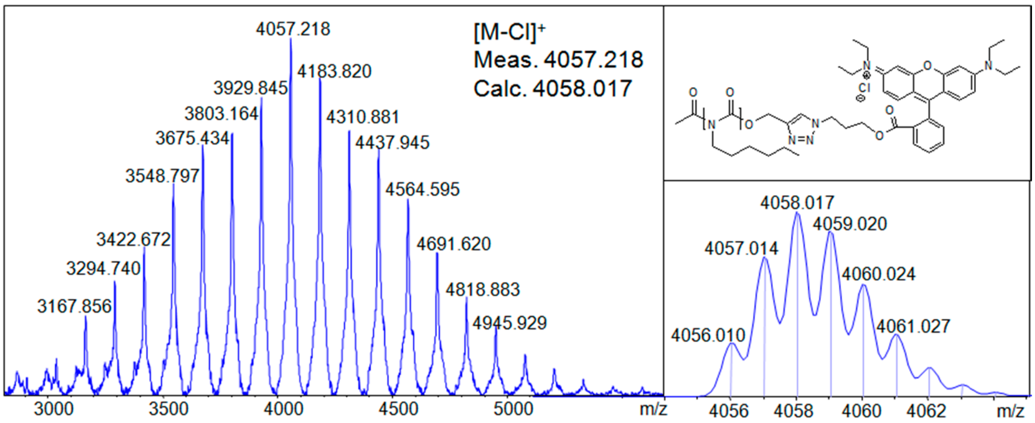

Figure S2. MALDI-TOF MS of Rh-PHIC.

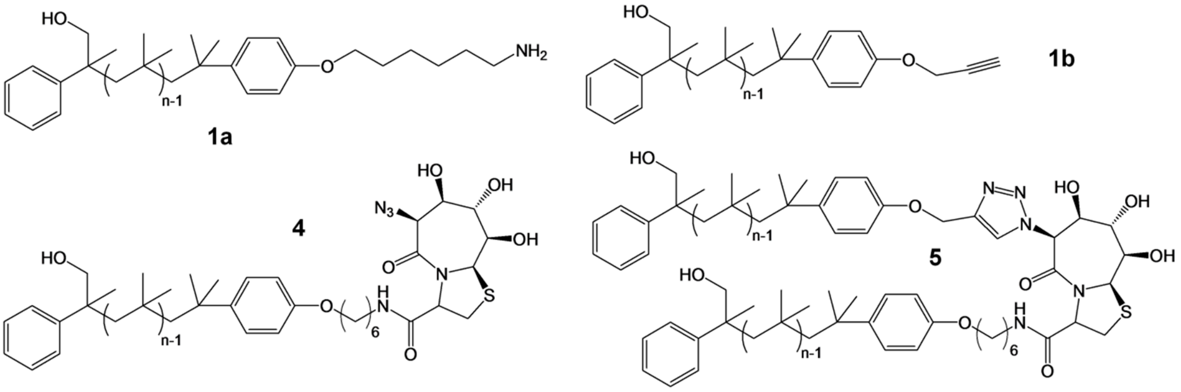

Figure S3. Chemical structures of PIB **1a** and **1b** and of PIB conjugates **4** and **5**.

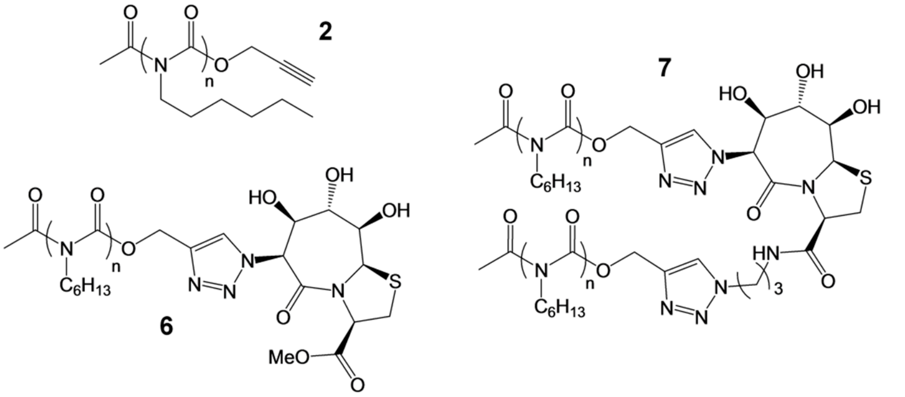

Figure S4. Chemical structures of PHIC **2** and of PHIC conjugates **6** and **7**.

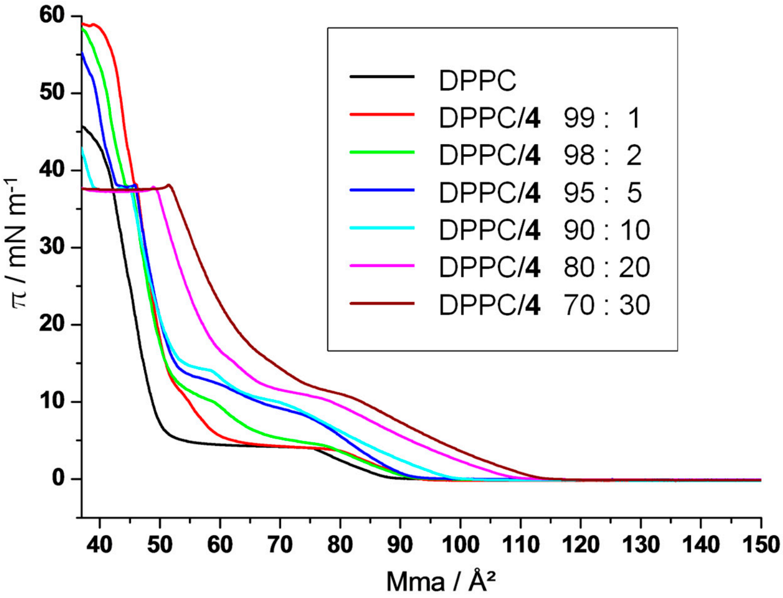

Figure S5. π-A isotherms of DPPC/**4** mixtures at 20 °C. The black curve represents the isotherm of the pure DPPC and the different colored curves represent the different molar ratios of the DPPC/polymer mixtures.

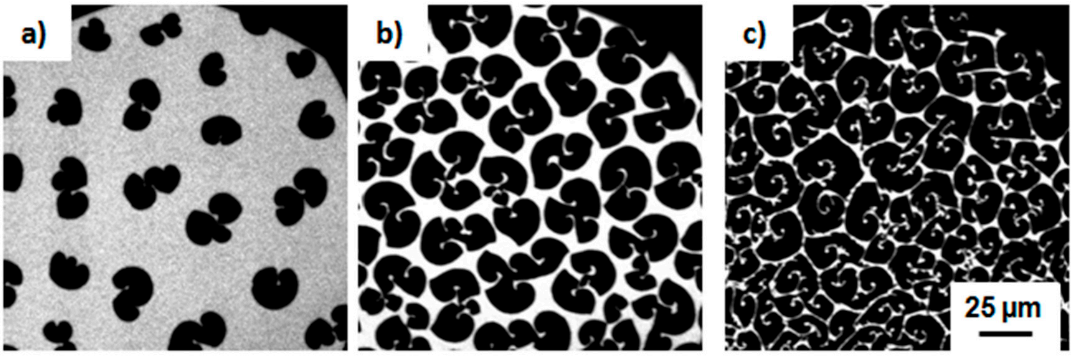

Figure S6. Epifluorescence microscopy images of L-DPPC monolayer at the air/water interface at 20°C. The images were recorded at constant compression of the spread at the following surface pressures: (a) 5.3, (b) 6.1, (c) 8.4 mN m^{-1}.

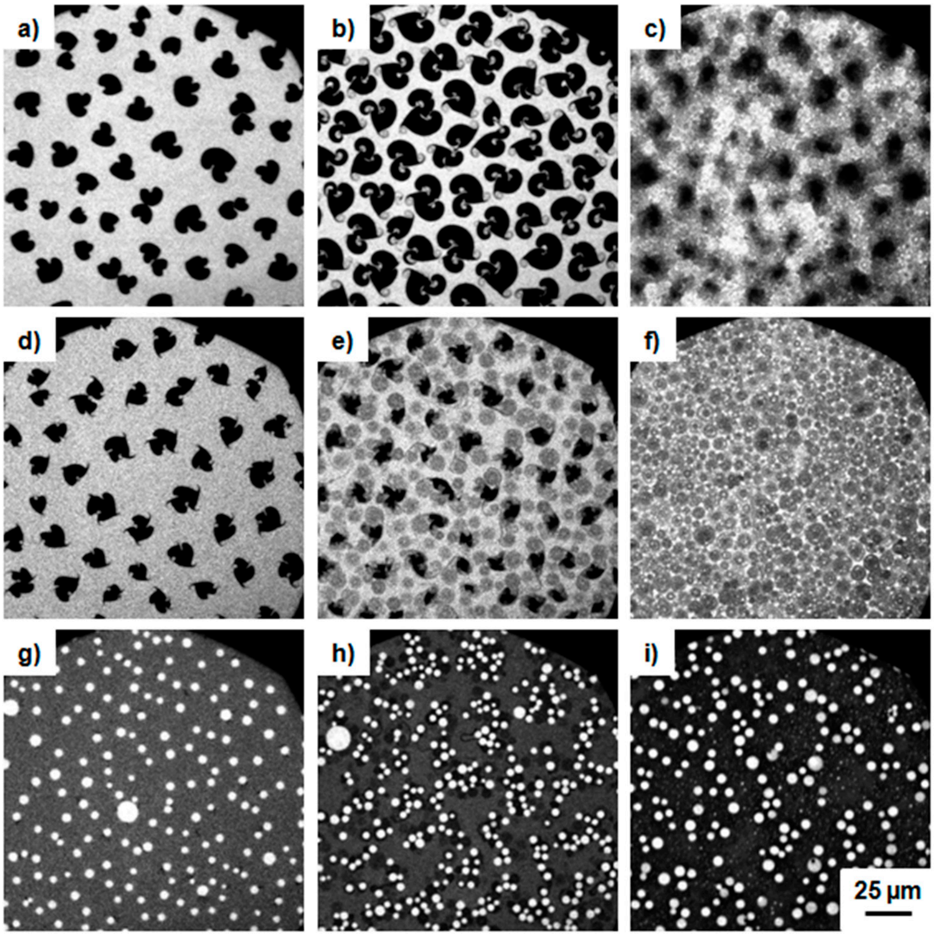

Figure S7. Epifluorescence microscopy images of monolayers of DPPC/**4** with different molar ratios (a-c) 99:1, (d-f) 98:2, and (g-i) 90:10 at the air/water interface at 20°C. The images were recorded at constant compression of the spread monolayer in the region of the second transition state (second plateau) at the following surface pressures: (a) 5.8, (b) 7.2, (c) 7.6, (d) 6.6, (e) 6.9, (f) 9.7, (g) 9.4, (h) 10.3 and (i) 29.2 mN m^{-1}.

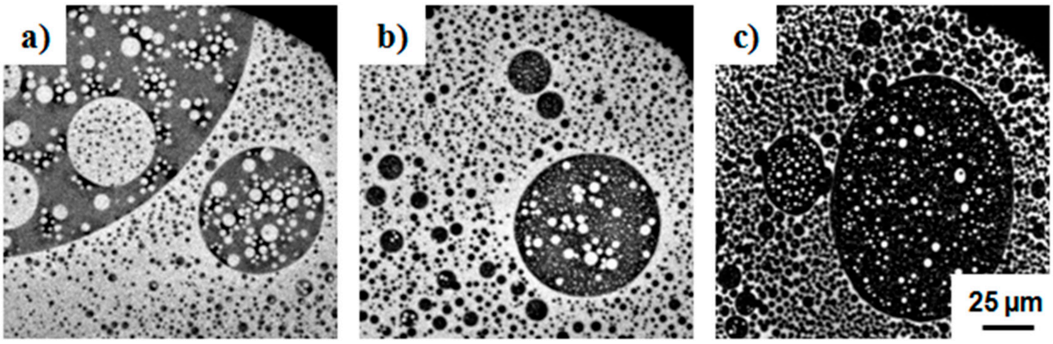

Figure S8. Epifluorescence microscopy images of monolayers of DPPC/**5** 80:20 at the air/water interface at 20°C. The images were recorded at constant compression of the spread monolayer at the following surface pressures: (a) 9.8, (b) 14.0, (c) 36.0 mN m^{-1}.

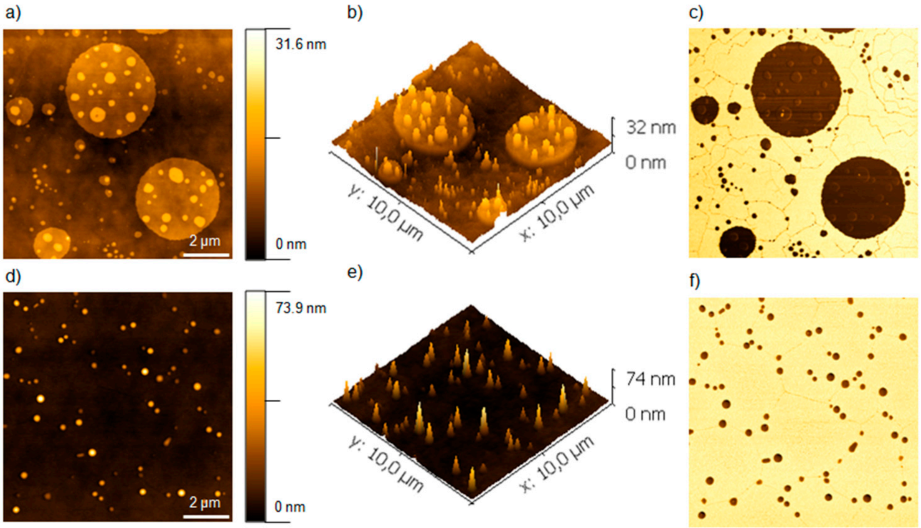

Figure S9. AFM topography (a, b, d and e) and phase contrast images (c and f) of mixed monolayer of DPPC/**4** 90:10 mol% transferred at a surface pressures of (a-c) 30 mN m^{-1} and (d-f) 40 mN m^{-1}, respectively.

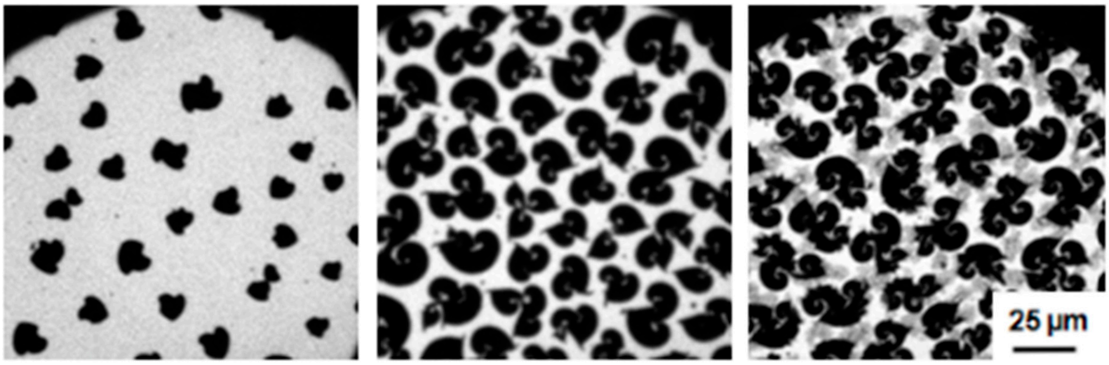

Figure S10. Epifluorescence microscopy images of monolayers of DPPC/**7** 99.5:0.5 at the air/water interface at 20°C. The images were recorded at constant compression of the spread monolayer at the following surface pressures: (a) 5.5, (b) 7.4, (c) 8.7 mN m^{-1}.

References:

1. Baier, G.; Siebert, J.M.; Landfester, K.; Musyanovych, A. Surface click reactions on polymeric nanocapsules for versatile functionalization. *Macromolecules* **2012**, *45*, 3419-3427.
2. Deike, S.; Binder, W.H. Induction of chirality in β-turn mimetic polymer conjugates via postpolymerization “click” coupling. *Macromolecules* **2017**, *50*, 2637-2644.
